# Balancing preterm infants’ developmental needs with parents’ readiness for skin-to-skin care: A phenomenological study

**DOI:** 10.3402/qhw.v8i0.21370

**Published:** 2013-07-11

**Authors:** Ingjerd Gåre Kymre, Terese Bondas

**Affiliations:** 1 Center for Practical Knowledge and Institute for Nursing and Health, PHS, University of Nordland/UiN, Bodø, Norway; 2 Institute for Nursing and Health, PHS, University of Nordland/UiN, Bodø, Norway

**Keywords:** Reflective lifeworld research, NICU nurses, Kangaroo Mother Care (KMC), Kangaroo Care (KC), proximity, skin-to-skin care (SSC), touch

## Abstract

The aim of this article is to articulate the essence and constituents of neonatal intensive care unit (NICU) nurses’ experiences in enacting skin-to-skin care (SSC) for preterm newborns and their parents. SSC is commonly employed in high-tech NICUs, which entails a movement from maternal–infant separation. Parents’ opportunities for performing the practice have been addressed to NICU staff, with attitude and environment having crucial influence. The study was carried out with a reflective lifeworld research approach. Data were collected in Denmark, Sweden, and Norway by open-dialogue interviews with a purposive sample of 18 NICU nurses to achieve the essence of and variation within the phenomenon. NICU nurses experience balancing what they consider preterm newborns’ current and developmental needs, with readiness in both parents for SSC. They share an experience of a change in the history of NICU care to increased focus on the meaning of proximity and touch for the infants’ development. The phenomenon of enacting SSC is characterized by a double focus with steady attention to signals from both parents and newborns. Thereby, a challenge emerges from the threshold of getting started as the catalyst to SSC.

Skin-to-skin care (SSC), meaning preterm infants cared for skin to skin to a parent's body, is commonly employed in high-tech neonatal intensive care units (NICUs), continuously or intermittently. The 7th International Workshop on Kangaroo Mother Care (KMC) recommends that it should begin as soon as possible after birth, be applied as continuous skin-to-skin contact to the extent that is possible and appropriate, and continue as long as appropriate (Nyqvist et al., 2010). Charpak & Ruiz (2011) consider SSC a component of the term KMC, which involves kangaroo position (KP); kangaroo nutrition, based on breast feeding; and mother–family involvement. The first introductions of the practice were inspired by the Instituto Materno Infantil in Colombia after 1978 (Charpak & Ruiz, 2011). KMC, or KC (Kangaroo Care), has been suggested as the foundation for a paradigm shift in neonatal care, from a movement from maternal–infant separation and from health care workers to parents as primary providers and caregivers (Ludington-Hoe, 2011; Nyqvist & Engvall, 2009; Nyqvist & Heineman, 2011), which we assume has influenced how NICU nurses have experienced their engagement in SSC. A policy survey in eight European countries on parental involvement and KC concluded that the ways, roles, and intensity of involvement by both parents varied in and between the countries, yet most units reported encouraging both parents to take part in the care (Pallás-Allonso et al., 2012).

Previous studies have addressed requests to NICU staff members to provide optimal conditions for parents to be with their child (Wigert, Berg, & Hellström, 2009), as well as offer opportunities for parents to be present and provide KMC for their infants to the extent that they are able and willing to do so, and as permitted by the infants’ medical condition and care (Blomqvist & Nyqvist, 2010). Parents’ opportunities for performing KMC have been addressed to the NICU staff, with attitude and environment having crucial influence (Blomqvist, Frölund, Rubertsson, & Nyqvist, 2012).

However, nurses describe their professional role in the NICU as diverse and demanding at the instrumental, emotional, and interpersonal levels (Fegran, Helseth, & Fagermoen, 2009). They found that creating a good relationship with parents always will put great demands on their personal attitude and capacity, and described interaction with parents as perhaps the most challenging part of their job.

Studies concerning SSC from the perspective of NICU nurses are limited, indicating there is a gap in the knowledge of the phenomenon of enacting SSC, to which focus on the lived experiences of nurses who influence the context might contribute.

The terms SSC, KMC, and KC will be used similarly to the referred articles, thus SSC is the term used in this study, to signal openness to the phenomenon, independent of procedural implementation.

## Aim

The aim of this article is to articulate the essence and its constituents of neonatal intensive care unit (NICU) nurses’ experiences in enacting SSC for preterm newborns and their parents.

## Method

The approach of reflective lifeworld research as developed by Dahlberg, Dahlberg& Nyström (2008) was carried out, which in its turn is based on the phenomenological philosophy of Husserl, Heidegger, Merleau-Ponty& Gadamer. The approach is an empirical application that is outlined by drawing on their philosophy of science (Dahlberg, Dahlberg, & Nyström, 2008, p. 25), which makes the lifeworld or the sphere of lived experience the starting point for all meaningful experience. Both phenomenology and hermeneutics within this approach want to grasp the meaning of phenomena.

To this article, a phenomenological descriptive analysis was chosen, which seeks to explore the essence of the phenomenon as faithfully as possible. The approach assumes an open attitude to the defined phenomenon, in this case the essence and its constituents of NICU nurses’ experiences in enacting SSC for preterm newborns and their parents. The phenomenon belongs to the context of a preterm newborn placed in KP, vertically skin to skin on a parent's chest, and the nurse in enacting and facilitating this practice. The open attitude is often described in terms of “bracketing,” or “bridling,” as used by Dahlberg, Dahlberg & Nyström (2008), which involves a willingness to listen, see, and understand to allow the deeper meaning of phenomena to come to expression. Within the realm of the lifeworld, phenomenology addresses itself to the constitution of experience.

The aim, according to Dahlberg, Dahlberg& Nyström (2008), is to describe, clarify, and elucidate the lived world in a way that expands our understanding of human being and human experience, and the clarification of meaning as it is given through their descriptions. The approach seeks for an essence and for its constituents that make the phenomenon that very phenomenon.

### Participants

A purposive sample of 18 nurses from three NICUs in the Scandinavian countries Sweden, Norway& Denmark (six from each) were interviewed at their workplace. Unit leaders were helpful in finding them from the criteria that they were willing to participate and available to be interviewed during two specific days and afternoons. Nurses with NICU practice for more than 5 years were prioritized to ensure practical experience. The Swedish nurses had practiced 3–24 years (median 13) in an NICU, the Norwegian 4–22 years (median 11), and the Danish 7–22 years (median 12). Twelve had a higher degree or education in paediatric, neonatal, intensive, surgery, or public health nursing; NIDCAP (Newborn Individualized Developmental Care and Assessment Program) education; or other specialized courses. NIDCAP is a theory-based caring model that focuses on detailed reading of each individual infant's behavioural cues to support and enhance each infant's strengths and self-regulation capacities (Als & McAnulty, 2011). Two nurses, who had practiced less than 5 years due to maternity leave, were included. All were female, although this was not a criterion. University NICUs confirming SSC as belonging to their practice were selected in anticipation that it was where the phenomenon existed. The sample represents both continuous and intermittent SSC practice; two units represent all preterm newborns, including extremely preterm, and one unit from the 28th gestational week. The Danish NICU was the oldest and most crowded, and the others were comparatively new. The Swedish unit had beds, the Norwegian and Danish, recliner chairs available for parents and preterm newborns for SSC. The choice to use informants from three Nordic countries was based on the assumption of a common history and culture of NICU care across these three countries. But in addition to significant common ground, the practices in Norway, Sweden, and Denmark would simultaneously provide sufficient variation and nuance to give a more comprehensive treatment of the phenomenon under investigation as well as to suggest elements of a Nordic perspective on SSC. Data collection in the three Scandinavian countries was not meant for comparison, but to achieve variations and nuances of experiences regarding the essence and meanings of the phenomenon.

### Data collection

The interviews were conducted in silent rooms separated from the NICU activity. Some short interruptions occurred, and some participants arrived directly from demanding work situations. They were prepared, were interested in participating, and had read the information letter beforehand. Two replaced others who were absent, but these also read through the information letters before conducting the interviews. They were asked to describe their lived experiences concerning their facilitating and enacting of SSC. The first author carried out the open-dialogue interviews, and asked them to tell about one particular or several self-experienced situations of SSC that they for some reason remembered especially, as well as common situations of acting and reasoning. To explore what they considered important, probing questions were asked to obtain details and to clarify unclear statements. In accord with reflective lifeworld research, their awareness and understanding of the context were articulated partly through dialogue with the interviewer. The interviews were conducted with an open attitude from the researcher in terms of bridling. The interviews took place in November and December 2009, and the digitally recorded material was transcribed verbatim by the first author during the spring of 2010. None of the authors had any connection to the NICUs.

### Ethical considerations

This study was approved by the regional committee for medical and health research ethics (REC), which carries out an assessment as to whether it is being undertaken in an acceptable manner (document reference: 2009/106-18). It is also approved by the data protection official for research, Social Science Data Services (NSD) (project number: 22199). The material was stored according to guidelines of the National Committee for Research Ethics in the Social Sciences and the Humanities (NESH, 2006) and ethical guidelines for nursing research in the Nordic countries (Northern Nurses Federation, 2003). Permissions to carry out the interviews, based on written information about the study, were obtained from the respective unit leaders in all countries. A letter from the first author introduced the participants to the aim of the interview. Permission to record the interviews was given from each participant, who was assured that the information would be treated confidentially. They were informed about their right to retreat from participating before they signed an approval.

### Analysis

A phenomenological method of analysis was chosen for this study, which aims at faithful description of the essential structure of the phenomenon. In line with Dahlberg, Dahlberg & Nyström (2008), the entire interview text was initially read to get a sense of its wholeness aspect. Meaning units in terms of smaller segments of the text were identified, which means dividing the whole into parts with respect to the meaning that was seen. It was important throughout to maintain an attitude of reflective distance to the material, a stance that is often referred to by phenomenologists as “bracketing” (Husserl) or “bridling” (Dahlberg). In this case, careful attention was paid to how the phenomenon and its meanings of enacting SSC were made explicit. Meanings that seemed to belong to each other were temporarily put together in clusters, then related to each other in looking for essential meanings and structures that describe the phenomenon. This part was conducted by always seeing the parts against the whole, and by being sensitive to nuances and changes in meaning. A new whole describes the phenomenon's essence, its essential meanings, and its structure of meanings in the context of NICU nurses enacting skin-to-skin contact between preterm newborns and their parents.

The presentation of the phenomenon is described in terms of the essence, followed by an identification of its constituents, which are the meanings that constitute the actual essence. Nuances that were present in the original data will be shown in the constituents.

Citations in the constituents are anonymous and marked with letters for Sweden (S), Denmark (D), and Norway (N), with numbers to indicate the participants.

## Findings

From the perspective of Scandinavian NICU nurses, the essential structure of meanings in enacting SSC is described as follows:

NICU nurses experience balancing what they consider preterm newborns’ current and developmental needs with how they meet parents’ readiness for SSC. The context was characterized by, first, their current double-focused steady attention to signs of readiness in parents and signs of comfort, stability, or instability in the preterm newborns; and, second, the double focus on the infants’ developmental prospects and the parents’ emotions and confidence in parenting.

The participants described how they perceive signs indicating that preterm newborns are more comfortable and well with SSC than alone in an incubator, and they focused on parents as the most important caregivers in giving them closeness and comfort through SSC. Thus, they consider that all parents are not necessarily ready for the first possible SSC sessions and need to be encouraged.

Awareness of a changing attitude in the nurses themselves characterized the descriptions, from a focus on caring for preterm newborns in incubators to a focus on attachment and offering parents more closeness to their newborns, and, even further, to the current attitude that is focusing on the preterm newborn's right and need to be close to its parents skin to skin for developmental purposes. However, the current attitude includes encouraging both parents with strength and understanding of themselves as the most important caregivers in that initiating SSC as soon as possible after birth, and as much as possible, is dependent on parents’ readiness for their necessary participatory presence.

On one hand, the nurses described having succeeded when both parents actively take part in the care and when they perceive harmony in witnessing the parent–child relationship in SSC. On the other hand, they consider it dependent on an active approach from the nurses themselves, especially in getting started, which was described as the critical threshold in order to achieve a harmonic SSC relationship. Perceiving and interpreting signs of readiness in parents influence their approach to SSC, and they are challenged in balancing how to share the responsibility of caring for preterm newborns with the parents.

### Shared experience of a changing focus in the history of NICU care

There was a shared experience of a changing focus in NICU caring practice from focus on the child in the incubator to attachment and parent focus, and further to an increasing focus on developmental issues and brain maturation in the infant. The nurses looked back in time when parents did not hold the infant outside the incubator for the first couple of weeks, and they remembered that they used to think “Hands off” to avoid stress in the infant, exemplified by the statement “We did not touch them if we did not have to” (D5).

Whether they focus on the present moment or on future outcomes, the focus on momentary wellness in the preterm is strong, as exemplified by the statement “The most important is the baby feeling good and next, the attachment is a bonus as well as the parents feeling the newborn is theirs” (S5). The quotation indicates a double focus, where the baby has the priority, and a current common experience is exemplified by the statement “When they get to know each other, they relax and parents can give something to the child that we cannot” (D4).

It can be challenging to prevent stress in infants from light and noise in crowded rooms, according to the nurses, with respect to their understanding of NIDCAP; thus, comfort on a parent's skin is preferred when trying to adhere to NIDCAP principals as well. A nurse said,

We have moved forward in NICU practice, in realizing the importance of attachment and the matter of perceiving each other skin to skin, and we know from studies that it is important for breastfeeding and for brain development. (D1)

### Initiating SSC as soon and as much as possible

Small preterms are normally intubated and cared for in incubators during the first days of life, but SSC is initiated as soon as possible: “As soon as it is secure and the baby can tolerate the transfer, we do it” (N3), one said. Thus, the findings indicate that what “as soon as possible” means depends on how the medical condition and safety are considered by individual nurses and physicians. Varied considerations of safety were expressed: *“*The sicker the newborn is, the more important it is to get started to have SSC, I think” (S1). In contrast, with some it was found safe to wait for more stability. Transfer of the smallest infants was described as the risky part and was secured by two nurses doing it together.

If mothers have had a caesarean delivery or are depressed, the nurses consider long SSC sessions during the first days hard for them, and they try to figure out the best solutions for each family, which often means SSC with fathers, who they have not always involved in this way: “We did not use to be good in acknowledging the importance that fathers have SSC from the beginning” (D1), one said. Even more often, fathers are the first to have SSC with their newborns, sometimes by carrying them skin to skin from the delivery room in a wrap. The nurses claimed that SSC makes fathers stay longer with the newborn because they feel needed.

The nurses have observed that parents find it natural to have SSC for hours if they do it from the beginning, and parents relieve each other, which the nurses recommend that they do as much as possible. Therefore, the point is that they feel it is important to do it as soon as possible.

### Signs of wellness and harmony are motivating for SSC

The nurses trust their sensibility of signs in preterm infants whether they feel comfortable, and attention to signals characterizes how they perceive the infant's condition: “I have seen they get more stable, and since studies have found the brain develops better, I understand its importance” (S1). They referred to signs of wellness, general stability, putting on weight, and a deeper state of sleep in regard to the infants, and the experience of success for mothers when their infant calms down skin to skin. Awareness of signals influences what nurses emphasize to parents to focus on.

SSC is experienced as a mark of turning or the only solution if the preterm shows stress. In contrast, “In moving him back into the incubator, you could see him perceive something was missing by his bodily unrest” (D2), a nurse said. Unstable preterms have responded positively to SSC by stabilizing. Those experiences strengthen the nurses’ beliefs that preterm infants get bodily stable as a result of SSC, which therefore motivates them to encourage its practice.

They focused on the experiences of proximity, exemplified by the following:

It is important that she dare attach to her child, feels like a mother, perceives him and learns to understand his signals, as well as he perceives his mother; it is the closest you can get outside the womb, I think. (D4)

SSC is also seen as a way to cope with the situation: “I see how they both relax, and I think it is something we easily can do in a situation that also contains disquieting information and experiences” (N5).

### Encouraging parents to dare to hold their newborn skin to skin

The nurses describe their approach to SSC as determined by how they perceive parents’ oral expressions, signals, and reactions in the first meetings with their newborns. Stress and anxiety in parents were expressed as being visible on their faces and bodies in terms of them being stiff as a poker and keeping a distance from the infant; and nurses can see on the whole body whether a parent manages to relax. The quotation “Stressed parents affect stress in the preterm newborn as well” (N6) expresses a challenging situation of establishing harmony in both parents and newborns. A nurse said,

If I manage to help the parents to relax and get confident to the SSC situation, the accounts are correct, if not it might be against its purposes and stressful for all, including the child. (N4)

Giving up SSC by transferring an uncomfortable newborn back into the incubator was seen as a failure in nursing care that possibly could make the parents more anxious.

Sometimes we persuade parents to hold their baby skin to skin, especially the smallest, who they don't dare to touch. We cannot push them too hard, but we may sometimes be impatient in getting started with SSC. The outcome is normally good, though we have to go step by step to get there. (S5)

They often see that parents are insecure and scared, with a tendency to think the newborn is safer and better off in the incubator, but “when they get started, they continue to do it every day” (N4).

The experience that some parents decline and therefore need to be encouraged or persuaded to hold and stay with the newborn is common. The nurses argued that SSC means improved infant developmental and future health, and that both brain and body develop better; they referred to documented effects, but mostly they referred to successful experienced situations.

The nurses experience it as challenging for parents to overcome the threshold of getting started, which is also a challenging trial of nurses’ patience on behalf of the baby. It is not unusual for the nurses to spend a couple of days preparing parents to hold the preterm child, according to the descriptions. They said some mothers are not well after birth, and fathers might not be available because of work or siblings they have to care for.

It is considered important to be sensitive and not make parents feel guilty for not being there continuously. Challenges were expressed due to what the nurses consider the child's needs and right to be comforted skin to skin, and due to how they encouraged parents to SSC – whether they should support, offer, or demand parents to dare to endure being with their child.

A problem in terms of parents’ willingness to endure SSC occasionally occurs in all units; the statement “Sometimes I persuade parents to stay for at least two hours if they get bored” (N4) expresses another aspect to the caring focus and exemplifies a variation to the phenomenon, yet is still a part of it.

### Acknowledging shared responsibility for the child

Encouraging parents towards SSC implies sharing knowledge with them, which in its turn implies shared responsibility in caring. The nurses have noticed that most parents become very sensitive to a baby's behaviour and pay attention to their baby continuously. The statement “ There is much you don't notice if you close the incubator doors, and there is another focus when they are skin to skin” (D2) reflects the common experience that parents perceive signals of stress and discomfort, which helps them quickly provide stability, and in responding early they prevent alarming monitors. The following statement additionally indicates the ascertainment of increased confidence in parents.

In my experiences, parents get enormously sensitive in having SSC, which also make them know, after some time when the preterm does not want SSC anymore. (N4)

The nurses, however, emphasized their responsibility in securing safety and stability for the preterm in SSC, which means they are still there to support parents and to watch infants’ medical condition. The nurses express an awareness of physiological instability as the nature of preterm infants, and they find it their responsibility to secure the SSC situation: “I pay attention, because there is often an incubator, monitors or lines to watch” (N5). Reflections on responsibility were connected to security, to preventing harm, and to the matter of responsibility for not leaving the child alone without skin-to-skin contact.

The participants recalled situations where parents were absent and NICU nurses stepped in to comfort the infants:

It might be excessive to have another woman's child on your skin, but I unbuttoned, and held him skin-to-skin, because it was his only possibility to have physical contact. (D2)

In another particular situation, nurses were hired to care for a child skin to skin. These initiatives were appreciated by the participants as an acknowledgment of giving priority to what they consider the child's rights and needs, and staff's responsibilities in comforting infants close to human bodies: “I believe it means a lot for their development and we might help them prevent cognitive and motor disorder” (D6).

## Discussion

### The concern of getting started

This study illuminates the essential meaning that NICU nurses experience when balancing what they consider preterm newborns’ current and developmental needs with how they meet both parents’ readiness for SSC. In spite of different grades in the implementation of SSC practice and different environmental conditions at the participating units, a joint concern about readiness in parents was illuminated as challenging. When the nurses consider the newborn ready for SSC, some of the parents might not yet be ready. That concern is double in that the shared experience of SSC for both the infant and the parents will be delayed. Even if research evidence considers SSC safe, as indicated by studies concerning thermal balance (Karlsson, Heinemann, Sjörs, Nyqvist, & Ågren, 2012; Maastrup & Greisen, 2010), the challenging threshold of readiness will most likely still exist in terms of what parents signal, and how the nurses perceive and react to their signals. In Flacking, Ewald, Nyqvist& Starrin (2006), some mothers did not dare to touch their infant, as they looked so fragile. In Skene, Franck, Curtis& Gerrish (2012), parents felt afraid of touching their infants during the first days, often related to a concern of causing harm and discomfort. Those studies exemplify situations that we assume nurses might still meet as normal reactions, making every new instance a new challenge of steady awareness and active approach, step by step.

Bigelow, Power, MacLellan-Peters, Alex& McDonald (2012) highlight another aspect to the concern of getting started with SSC with the finding that it benefits mothers by reducing their depressive symptoms and physiological stress in the postpartum period. Eriksson & Pehrsson (2005, p. 134) describe parents of extremely low-birth-weight infants’ situation as “particularly difficult because, (…), the parents are in a chaotic emotional state at the same time as they are supposed to meet a very small (and sometimes very ill) child.” From their research on parents participating in a care and psychosocial support programme, they found emotional confusion and varying levels of fear, anxiety, anger, sadness, and joy. Those findings cohere to the lived experiences of nurses from this study, who perceives varying emotional levels in parents, which influences how they interpret and meet their readiness for SSC. The findings in this study indicate an importance in reflecting on how nurses encourage parents to participate and feel needed as a condition for the developmental care of their newborn. In births of newborns in general, new-life situations are brought forth, and in Bondas (2005) the women expressed that they needed guidance and advice that had a base in knowledge, not in trial and error. Also, Nyqvist et al. (2010, p. 823) emphasizes the positive aspect of how the concept of KMC “enhances parent-infant bonding and attachment and transforms the crisis of having a preterm or ill newborn infant into a more gratifying experience for the whole family.” The way that nurses in this study focus on how they perceive and encourage parents’ approach to the newborns signalled an embedded comprehension that parents need to comfort and hold their child, and a concern on behalf of those who were reserved. This emphasizes how the context is strongly influenced by perceptual awareness in each particular instance, expressed within an acknowledgment of the significance of a nurse's active approach to SSC. Seeing that a challenge emerges from the threshold of getting started as the nexus or catalyst to SSC and the movement against confidence in parents to endure SSC, a reflection on what is at stake is essential when discussing the essential meaning structure of the phenomenon.

### The core of what is at stake

The participants expressed an awareness of increased inward focus with both a preterm infant's current and developmental needs at stake, which they attempt to achieve through SSC. The findings are in line with Fegran, Helseth& Slettebø (2006, p. 62), who found that nurses caring for hospitalized premature children have experienced a radical change in the philosophy of care: “From being concerned primarily with a child's physiological condition, nurses today are strongly aware of a child's various and complex needs, not least the importance of child-parent attachment.” The findings in this study indicate that the experienced change has moved even further against an increased focus on the developmental needs in preterm infants, which some participants addressed to NIDCAP theory. The findings are also in line with the study concerning what it is like to be a neonatal nurse after developmental care is introduced to the unit, by Hall, Kronborg, Aagaard& Ammentorp (2010). In their study, the responsibility for the neonates and the developmental care of the baby was considered intertwined with empowering parenting, which was a central part of nurses’ daily work; however, the nurses put caring of the infant as primary. Those similar findings remind us that how SSC is focused by nurses reflects an influence and intertwining with other NICU intervention models of care, which nurses in this study express through how they focus, and what they focus on that belongs to the phenomenon. Their superior focus indicates that nurses consider parent participation both as conditioning a co-functioning relationship with the preterm for the welfare of both as a unit and as necessary instruments for the child's developmental needs. The connection between NICU intervention models has been emphasized in research, notably Als and McAnulty (2011), who sees SSC as a component of NIDCAP.

Encouraging fathers towards SSC soon after birth improves the situation for infants by not having them wait for SSC. Blomqvist, Rubertsson, Kylberg, Jöreskog& Nyqvist (2011) found KMC gave fathers increased responsibility for the infants’ care, and Fegran, Helseth& Fagermoen (2008) found fathers’ early involvement to be “strengthened by their positive skin-to-skin experiences and by the mothers encouraging them as important contributors to the child's care.” Bondas (2005) found partners’ presence meant communion and creating of their families in women after normal birth situations, which we suggest might be equal to a preterm birth situation and therefore needs attention.

The nurses in this study considered that parents become very sensitive to their infants’ behaviour in terms of alertness to signs and early responses in SSC, but they emphasized nurses’ responsibility in securing safety and stability for the preterm. They described experiences like those found in Skene et al. (2012, p. 11), in which parents “developed a unique knowledge of their own infants and used it to provide comfort in an appropriate way.” Johnson (2007) found that confidence in mothers in knowing how to respond to their infants “makes each holding better than the last,” which reflects the first SSC as a catalyst for the harmonic relationship.

The expressed challenge in sharing responsibility reflects a problem of not having full direct access to the child's condition. Thus, whereas responsibility in securing both safety and the SSC experience is given priority, nurses signal a challenge in themselves that they cannot avoid, which makes their steady attention to signals a necessary condition for balancing SSC. They signalled a deep commitment to wellness, in terms of providing the best developmental conditions for the child's maturing brain, which illuminates what we consider to be the core of what is at stake in how the participants enact and reflect on SSC.

Ludington-Hoe (2011) emphasizes the effects of the actions of mothers and the way in which these can contribute to and promote physiological stability, and have a positive influence on infant brain development by providing appropriate sensory experiences. Also, Milgrom et al. (2010) claim that the quality of early experience influences cerebral development, and Als & McAnulty (2011) highlight the concept of co-regulation from an evolutionary perspective. In addition, Tessier et al. (2003) emphasize the intertwined learning that SSC “potentiates the neurobiological development of the brain” and that “the physical proximity experienced by the parents gives them confidence in their infants capacities and makes them aware of the infants needs,” and that “these two components combine to favor the infants’ mental development.”

That knowledge makes the challenge in this study even clearer, inasmuch as we can see that the responsibility at stake in such situations has both ethical and developmental aspects connected to it as constituent factors. In this article, the challenge comes to a head against the experienced commitment of meeting varied resistance and fear in parents to hold the preterm newborn skin to skin when the infant is considered ready for it with both current and developmental purposes and prospects at stake.

## Conclusion

This article has illuminated the essence and its constituents of NICU nurses’ experiences of enacting SSC, which they experience as balancing what they consider preterm newborns’ current and developmental needs, with the readiness in both parents for SSC. They share an experience of a change in the history of NICU care to increased focus on the meaning of proximity and touch for the infants’ development. The phenomenon of enacting SSC is characterized by a double focus with steady attention to signals from both parents and newborns. Thereby, a challenge emerges from the threshold of getting started as the catalyst to SSC.

More research is needed on the conditions for willingness in parents to endure being with their infant, intertwined with a debate on the developmental aspect of SSC and how that knowledge is recognized in the care of preterm newborn infants in general.
